# Clozapine Intoxication Mimicking Acute Stroke

**DOI:** 10.5811/cpcem.2018.1.36734

**Published:** 2018-04-05

**Authors:** Jacob A. Lebin, Joshua D. Villarreal, Betty C. Chen, M. Kennedy Hall

**Affiliations:** * University of Washington, Department of Emergency Medicine, Seattle, Washington; † University of Washington, Department of Pharmacy, Seattle, Washington

## Abstract

Clozapine is an atypical antipsychotic drug prescribed for treatment-resistant schizophrenia. The risk of adverse hematologic, cardiovascular, and neurologic effects has tempered its use, and reports of overdoses remain rare. We report a case of accidental acute clozapine intoxication in a clozapine-naïve patient, who presented with symptoms mimicking acute stroke and later developed status epilepticus. Clozapine intoxication is a rare presentation in the emergency department with potential for iatrogenic harm if not correctly identified.

## INTRODUCTION

Clozapine is an atypical antipsychotic drug used for treatment-resistant schizophrenia and is the prototypical agent of the tricyclic dibenzodiazepine class. Atypical antipsychotics are effective in controlling the positive (hallucinations, delusions) and negative (flat affect, anhedonia) symptoms of schizophrenia with fewer extrapyramidal side effects compared to typical antipsychotics.^1^ In addition to affinity for the dopamine (D_2_) receptor, the unique clinical and side-effect profile of clozapine is mediated through mixed antagonism at muscarinic, histamine, alpha-adrenergic, gamma-aminobutryic acid, and serotonin receptors.

Despite its clinical superiority in comparison to other atypical antipsychotics, clozapine remains a second-line agent due to its side-effect profile.^1^ At standard doses, clozapine has been known to cause agranulocytosis, sedation, and hypersalivation.^2^ At toxic doses, clozapine has been reported to cause encephalopathy, dysarthria, and ataxia.^3^ Previously published reports of clozapine overdose have mainly been characterized by large-dose ingestions in patients who are attempting suicide and are already maintained on clozapine.^3^,^4^ There are few reported cases describing acute clozapine intoxication in clozapine-naïve patients with relatively small exposures.^5^

We report a case of a clozapine-naïve man who presented to the emergency department (ED) with dysarthria and ataxia, initially thought to be due to an acute ischemic stroke, but ultimately determined to be due to acute clozapine intoxication. Although uncommon, clozapine intoxication should be considered for patients who present with acute onset of neurologic symptoms and possible clozapine exposure.

## CASE REPORT

A 66-year-old man with a history of hypertension and hyperlipidemia was brought to the ED for altered mental status. The patient’s wife reported that two hours prior to arrival, he had ingested two glasses of wine and took a nap. Prior to napping the wife noted no unusual symptoms. Upon awaking, he was noted to have dysarthria and discoordination of his extremities and was brought to the ED. His wife reported that his home medications included bupropion, dutasteride, lisinopril, and tolterodine.

His vital signs were blood pressure 115/78 millimeters mercury, heart rate 83 beats per minute, respiratory rate 16 breaths per minute, temperature 36.8° Celsius, and oxygen saturation of 95% on room air. On exam, the patient was noted to have waxing and waning alertness, but he could intermittently answer questions and follow commands. His pupils were reactive, sluggish, and with horizontal and vertical nystagmus. His face was symmetric and his tongue was midline. His speech was dysarthric. He was unable to follow commands for strength testing, but he was moving all extremities without appreciable asymmetry. He had bilateral dysmetria on finger-to-nose and heel-to-shin testing. He had truncal ataxia in the seated position. His mental status and ataxia progressively worsened during his ED stay of approximately 1.5 hours.

Given the acute onset of symptoms, an institutional stroke alert was activated and a head computed tomography angiogram was obtained, which was negative for hemorrhage or other abnormality. Laboratory values, including complete blood count, chemistry panel, coagulation panel, and urinalysis were within normal limits. His blood alcohol level was 57 mg/dL. Electrocardiogram showed normal sinus rhythm with a QRS of 108 ms and a QTc of 497 ms. A dose of naloxone 0.4 milligrams was trialed without improvement in patient’s somnolence. He was evaluated by the neurology consultant team and determined to have a National Institute of Health Stroke Scale of 11. Given concern for a posterior circulation stroke, tissue plasminogen activator was administered for thrombolysis. The patient was subsequently intubated for airway protection due to increasing somnolence and admitted to the intensive care unit (ICU).

While intubated in the ICU, the patient developed status epilepticus, characterized by three periods of generalized tonic clonic activity, each lasting approximately five minutes. He received multiple doses of benzodiazepine and phenytoin, in addition to the propofol infusion for sedation, resulting in cessation of seizure activity. Subsequent electroencephalogram did not demonstrate ongoing seizure activity, and the patient did not have an additional seizure during the hospital course while maintained on phenytoin.

Magnetic resonance imaging of the brain did not show evidence of acute stroke. Comprehensive urine drug screen by gas chromatography and mass spectrometry was positive for clozapine and clozapine metabolites, in addition to caffeine and bupropion, a home medication. No drugs of abuse or other medications were detected. However, the patient had never been prescribed clozapine. In discussion with his wife, it was learned that the patient’s family member takes clozapine for a psychiatric condition. The patient manages the family member’s medication and they frequently take their respective medications at the same time. It was concluded that the patient must have mistaken the family member’s medication for his and accidently ingested the family member’s usual dose of 200 mg of clozapine, hours prior to his presentation. The patient was extubated on hospital day three and discharged with a normal neurologic exam and no further seizure activity on hospital day five.

## DISCUSSION

We present a case of acute clozapine intoxication in a clozapine-naïve patient. This case illustrates the features of clozapine toxicity and highlights that clozapine ingestions can lead to severe intoxications in naïve patients.

To our knowledge, this case represents one of the few reported clozapine overdoses in a clozapine-naïve patient.^5^ Patients beginning treatment with clozapine are usually started on 12.5mg daily with up-titration to daily doses of 300 to 600 mg. With our patient taking an acute ingestion of 200 mg, this case illustrates a severe intoxication at the lower end of usual therapeutic dosing. It has been postulated that tolerance may develop with prolonged clozapine treatment, resulting in more severe intoxications in those patients who have not been exposed to clozapine previously.^5^ In a retrospective case study of poison center reports, clozapine doses as low as 100mg were found to have resulted in severe intoxication, but clozapine pretreatment information was limited.^3^ It is important for providers to recognize that severe intoxications can occur even at standard clozapine doses.

**CPC-EM Capsule**

What do we already know about this clinical entity?

Clozapine is an atypical antipsychotic drug used for treatment-resistant schizophrenia. Reported cases of intoxication are frequently characterized by large-dose ingestions.

What makes this presentation of disease reportable?

This case illustrates a severe clozapine intoxication from standard dosing in a clozapine-naïve patient and was characterized by stroke-like symptoms and status epilepticus.

What is the major learning point?

Clozapine intoxication is a rare presentation to the emergency department. Emergency physicians (EP) should be familiar with the medication and its presentation in overdose.

How might this improve emergency medicine practice?

EPs should maintain a broad differential for stroke-like symptoms, including medications, and recognize the signs of clozapine intoxication.

The symptoms of acute clozapine intoxication presented here are consistent with those reported previously in the literature. Central nervous system depression, sometimes severe (Glasgow Coma Scale less than 7), is the most frequently observed symptom and is likely mediated through antagonism of the histamine H_1_ receptor.^3^ Patients also frequently present with dysarthria, bradykinesia, and ataxia. Due to clozapine’s partial antagonism at the muscarinic receptor, poisoned patients can present with symptoms of an anticholinergic toxidrome, such as tachycardia, altered mental status, and coma. Consistent with this case, patients over the age of 50 are at increased risk for severe intoxication.^3^

This case represents one of the few reported clozapine intoxications complicated by status epilepticus.^3^ Clozapine is known to lower the seizure threshold, possibly through antagonism of the gamma**-**aminobutyric acid receptor, and seizures have been reported during treatment and in overdose previously.^6^ However, reports of status epilepticus remain rare in the literature. It is likely that the patient was at higher risk for seizure activity given his use of bupropion, and thus had a lower seizure threshold prior to his presentation. An alcohol-withdrawal seizure was thought to be less likely as the patient did not have a history of heavy alcohol use or alcohol withdrawal, nor did he demonstrate symptoms consistent with alcohol withdrawal. It is also possible that the patient’s abnormal movements were the result of a dystonic reaction, but this would be unusual given the relative low affinity for D_2_ antagonism of clozapine as compared to typical antipsychotics.

Stroke mimics are common in the ED and account for up to 30% of suspected stroke presentations.^9^ Medications are a rare etiology of stroke mimic, which has only been described previously in case reports of methyl iodine and prochlorperazine.^10^,^11^ Presentations that mimic posterior circulation strokes are particularly challenging as the symptoms of posterior circulation stroke, such as ataxia, dizziness, and dysarthria, are shared with many alternative etiologies.^12^ Given the need for rapid decision-making in acute ischemic stroke, misdiagnosis and inappropriate treatment with thrombolytic therapy have become increasing common. Fortunately, the incidence of symptomatic intracranial hemorrhage in patients who received thrombolytic therapy for a stroke mimic remains low (0.5%).^9^ Our patient did not experience any adverse effects from the administration of tissue plasminogen activator.

Primary management of acute clozapine overdose is supportive care. Providers should be prepared to provide airway management given the frequency of somnolence and coma. Hypotension refractory to fluids may be present due to alpha-adrenergic blockade, and vasopressor support may be necessary.^5^ Quantitative clozapine levels require gas or liquid chromatography and are rarely available during early management.^4^ Therefore, the diagnosis of acute clozapine intoxication relies heavily on supportive collateral history of clozapine exposure, which can be challenging to obtain unless solicited.

## CONCLUSION

Acute clozapine intoxication is a rare presentation to the ED and is characterized by encephalopathy, dysarthria, and ataxia. Clozapine-naïve patients may be at higher risk for severe intoxication from standard dosing regimens.

Documented patient informed consent and/or Institutional Review Board approval has been obtained and filed for publication of this case report.
